# Metabolic Fate of the Increased Yeast Amino Acid Uptake Subsequent to Catabolite Derepression

**DOI:** 10.1155/2013/461901

**Published:** 2013-02-04

**Authors:** John S. Hothersall, Aamir Ahmed

**Affiliations:** The Institute of Urology and Nephrology, University College London, Charles Bell House, 67 Riding House Street, London W1W 7EY, UK

## Abstract

Catabolite repression (CCR) regulates amino acid permeases in *Saccharomyces cerevisiae* via a TOR-kinase mediated mechanism. When glucose, the preferred fuel in *S. cerevisiae*, is substituted by galactose, amino acid uptake is increased. Here we have assessed the contribution and metabolic significance of this surfeit of amino acid in yeast undergoing catabolite derepression (CDR). L-[U-^14^C]leucine oxidation was increased 15 ± 1 fold in wild type (WT) strain grown in galactose compared to glucose. Under CDR, leucine oxidation was (i) proportional to uptake, as demonstrated by decreased uptake and oxidation of leucine in strains deleted of major leucine permeases and (ii) entirely dependent upon the TCA cycle, as cytochrome c1 (Cyt1) deleted strains could not grow in galactose. A regulator of amino acid carbon entry into the TCA cycle, branched chain ketoacid dehydrogenase, was also increased 29 ± 3 fold under CCR in WT strain. Protein expression of key TCA cycle enzymes, citrate synthase (Cs), and Cyt1 was increased during CDR. In summary, CDR upregulation of amino acid uptake is accompanied by increased utilization of amino acids for yeast growth. The mechanism for this is likely to be an increase in protein expression of key regulators of the TCA cycle.

## 1. Introduction

In *S. cerevisiae* and other yeast, growth in glucose as the carbon source represses transcription of numerous genes (termed glucose repression or carbon catabolite repression, CCR) [1, 2]. Yeast grown with alternate carbon source (e.g., galactose or glycerol) undergoes catabolite derepression. During catabolite derepression (a condition akin to metabolic stress) yeast metabolism shifts from fermentative to respiratory and carbon is shunted to the mitochondrial TCA cycle thus increasing electron transport and respiration [3, 4]. 

We recently reported that during catabolite derepression (with galactose or glycerol as carbon source), there is an increase in yeast amino acid permease gene and protein expression, amino acid uptake, and oxygen consumption [5]. We further demonstrated that the signalling involved in the coordination of this process, via TOR1 [5], a phenomenon that is distinct from that involved in diauxic shift, the recurring life cycle in the natural history of yeast [6]. It has also been known for some time that synthesis of respiratory enzymes is increased in the presence of galactose compared to glucose [7]. Based upon these observations we put forward a *prima facie* model [5] suggesting that the surfeit of amino acids during catabolite derepression in yeast may serve as reductive substrates for the TCA cycle providing an electron source for mitochondrial energy transduction. Little information exists on the utilization of the increased amino acids under conditions of stress such as catabolite derepression in yeast.

In this paper we have tested our proposed model [5] by addressing the following question regarding the metabolic fate of increased uptake of amino acids under catabolite derepression: are the accumulated amino acids surplus to the altered metabolic requirement during catabolite derepression or do they serve as a fuel in biochemical pathways related to the observed increase in oxygen consumption? We used the WT yeast and strains with genetic alterations grown under CCR and catabolite derepressed conditions to investigate whether the increase in amino acid uptake during catabolite derepression is accompanied by (i) increase in the oxidation of amino acids and (ii) increase in the activity and expression of TCA cycle enzymes. Our results show a direct corelation between the increase in amino acid uptake and an increase in the trans- and deamination of amino acids during catabolite derepression accompanied by oxidative cell metabolism and increase in the TCA cycle turn-over during catabolite derepression. Furthermore, this increase in the TCA cycle turn-over is accompanied by an increase in the activity and protein expression of key TCA cycle and amino acid metabolizing enzymes. We demonstrate that the TOR mediated increase in amino acid uptake serves as a fuel for energy provision, thus providing a mechanism of yeast metabolic adaptation during conditions of stress. 

## 2. Materials and Methods

### 2.1. Materials

L-[U-^14^C]leucine (10.9 GBq/mmol) was purchased from Amersham Pharmacia Biotech, UK. Nitrocelluose filters (0.45 *μ*m pore size) were purchased from Whatman, UK, or Millipore, UK. Chemicals were obtained from Merck, UK, unless otherwise stated.

### 2.2. Yeast Strains and Growth Conditions

Wild type *S. cerevisiae* strains S288C variant M3750 (*MAT*a*ura3*; [8]), BY4741 (*MAT*a*hisΔ3*1 *leu2Δ*0 *met15Δ*0 *ura3Δ*0; purchased from the Yeast Knockout Collection (YKO) [9] through Open Biosystems, USA), and EY0986 (*MAT*a* his3Δ1 leu2Δ0 met15Δ0 ura3Δ0 *[10] purchased from the Yeast-GFP clone collection, Invitrogen, UK) were used in this study. Amino acid permease deleted yeast has been described earlier [11] and details of the genotype of the amino acid permease deleted strains used in this study are given in Supplementary Table 1 (see Supplementary Material available online at http://dx.doi.org/10.1155/2013/461901). Growth conditions are detailed elsewhere [5]. Oxygen consumption and L-[U-^14^C]leucine uptake were measured as described previously [5]. Glucose concentration in the culture supernatant of O/N yeast cultures was measured using a chemiluminescence assay [12] and was found to be 4.2 ± 0.3 mM (*n* = 3). For some experiments, yeast was grown on solid medium by adding 2% select agar (Invitrogen) to the YNB medium plus the carbohydrate source described above.

### 2.3. Leucine Oxidation

Leucine is a common substrate for all the neutral amino acid permeases upregulated during catabolite derepression under the control of TOR [5]. We therefore used L-[U-^14^C]leucine as a paradigm substrate for amino acid oxidation. L-[U-^14^C]leucine oxidation assay is similar to that described previously [13, 14]. Briefly, O/N cultures were harvested by centrifugation at 1000 ×g for 10 min at room temperature and washed with YNB medium without carbohydrates. Care was taken to remove all the media and the cell pellet was washed and resuspended in fresh YNB-CSM (without L-leucine) medium containing either glucose or galactose. In some instances carbohydrate substrate was omitted and in others the nonmetabolizable analogue of glucose, namely, 2-deoxyglucose (DOG), was used. Each corresponding suspension (5 mL) was added to L-[U-^14^C]leucine (750 *μ*M, 94 *μ*moles/*μ*Ci) in a 50 mL Erlenmeyer flask fitted with a 2 mL centre well, sealed with silicone rubber Suba seals, and placed in an orbital shaking incubator at 30°C (120 rpm). After 1 h the reaction was stopped by injecting through the Suba seal 200 *μ*L 5 N HCl into the flask and into the centre well 500 *μ*L of hyamine hydroxide (1.0 M in methanol, Packard (Caversham, Berks, UK). The flasks were returned to the incubator for a further 1 h to equilibrate ^14^CO_2_ absorption into the hyamine. The contents of the centre well were transferred to a scintillation vial together with the results of a 1 mL wash with methanol. Corresponding blanks without yeast were used to measure L-[U-^14^C]leucine background release of ^14^CO_2_ and a sample from the flask was taken for specific activity calculation. Samples were counted in a Beckman LS6000 liquid scintillation counter (Beckman-Coulter, UK).

### 2.4. Amino Acid Transport Assay

Yeast cells were recovered by centrifugation, washed in 0.6 mL of a HEPES based transport buffer [5], and finally resuspended to OD_600_ = 0.7 − 0.8. Radio-labelled amino acid uptake was measured using techniques described previously [5, 15].

### 2.5. Enzyme Assay

The basal activity of branched-chain *α*-keto acid dehydrogenase (BckDH, E.C.1.2.4.4) was measured in cell homogenates from yeast. The assay medium (pH 7.4) contained 30 mM potassium phosphate, 2 mM DTT, 5 mM MgCl_2_, 3 mM NAD^+^, 0.4 mM reduced CoA, thiamine pyrophosphate, and 0.05% (v/v) Triton X-100. Nonspecific reductive activity was assessed at 340 nm in a Beckman DU650 spectrophotometer (Beckman-Coulter, UK). Specific BCKD activity was measured upon addition of 0.5 mM *α*-ketoisovalerate. Activity was calculated from NADH formed using an extinction coefficient (Σ_340_) of 6220 M^−1^ cm^−1^. Intracellular glutamate was measured using a NADH/glutamate dehydrogenase linked assay in hydralazine buffer pH 9.0 according to the method of Bergmeyer [16].

### 2.6. Direct Fluorescence Microscopy of Green Fluorescent Protein (GFP) Tagged Yeast Strains

Yeast with GFP-tagged cytochrome c1 (Cyt1, YOR065W), citrate synthase (Cs, YNR001C), and dihydrolipoamide dehydrogenase (Lpd1, YFL018C) were grown either in glucose or galactose containing media. Fluorescence for Cyt1-GFP and Cs-GFP was performed using a Zeiss LSM510 confocal system (Zeiss, Germany) with an argon laser line (488 nm) and a transmitted light detector for bright-field images or a Bio-Rad Radiance 2100 confocal system (Bio-Rad) coupled to a Nikon TE300 inverted microscope equipped with a Nikon 60x oil-immersion lens (numerical aperture = 1.4) with an argon laser line (488 nm) and a transmitted light detector for bright-field images and analysed using ImageJ software (http://rsb.info.nih.gov/ij/). Experiments were performed in at least 3 different preparations with triplicate or quadruplicate measurements. Results are expressed as means ± SEM. Statistical analysis, where applicable, was performed using a Student's *t*-test. 

## 3. Results

**Figure 1:**
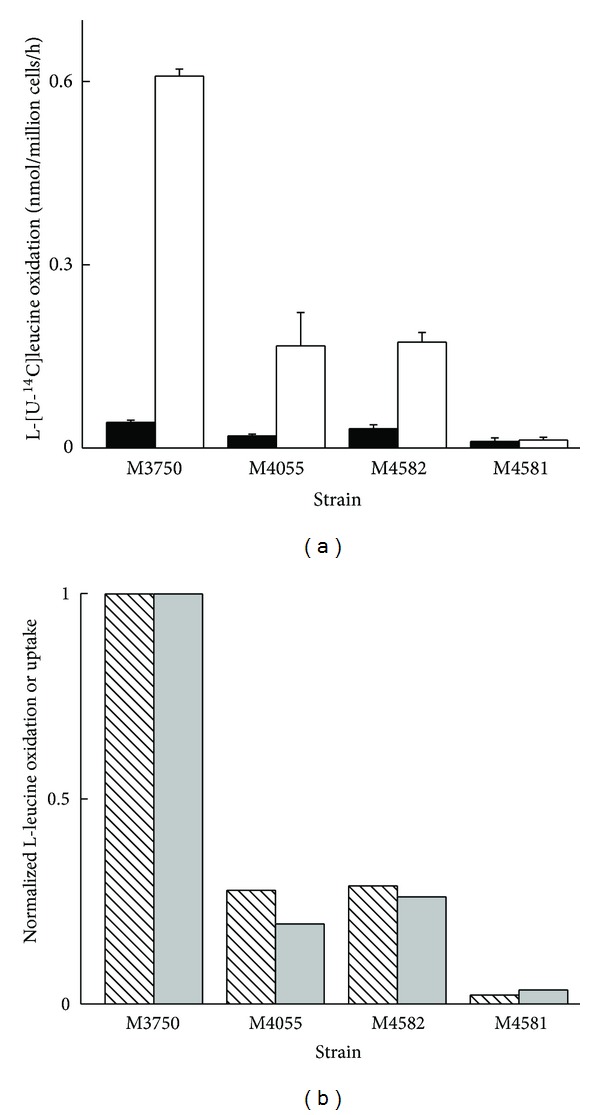
(a) L-[^14^C]leucine (750 *μ*M) oxidation in WT and amino acid permease deleted yeast strains. Yeast was grown in either 2% glucose (filled bars) or 2% galactose (hollow bars). L-[^14^C]leucine uptake and oxidation were performed as described in Materials and Methods. Deletion of various amino acid permeases (M4055 : MATa ura3 gap1 D D(bap2-tat1), M4581: MATa ura3 gap1D agp1D gnp1D D(bap2-tat1), M4582: MATa ura3 gap1D D(bap2-tat1) bap3D tat2D) from the WT (M3750) reduced the rate of L-[^14^C]leucine oxidation observed when yeast are grown in galactose. Results are mean ± SEM from 3 different preparations each measured in quadruplicate. (b) Correlation between carbon catabolite derepressed L-leucine uptake (shaded bars) and oxidation (hatched bars). For correlation analysis data (nmol/million cells/h), oxidation (0.7 ± 0.02) and uptake (1.2 ± 0.12) in the WT M3750 strain were normalized to 1. In deleted strains bars represent the proportion of either uptake or oxidation observed in the WT strain.

There was a large increase in the L-leucine uptake and also in the oxygen consumption in yeast grown under catabolite derepression, as reported previously [5]. There was a 16.6 ± 0.9-fold increase in L-[U-^14^C]leucine oxidation in yeast undergoing catabolite derepression than those grown in glucose under CCR (Figure 1(a)). The accompanying 15-fold increase in the oxygen consumption in the WT yeast grown in galactose compared to glucose medium, we have reported previously [5], was also observed for other WT strains used in this study (data not shown).

Is the pool of oxidized leucine derived largely from the increased L-leucine uptake during catabolite derepression? If the source of oxidized leucine was largely derived from the increased uptake, there should be a decrease in the leucine oxidation in yeast from which major amino acid permeases were deleted. The rate of leucine oxidation was proportionally decreased in strains from which the major TOR regulated amino acid permeases BAP1, TAT1, and GNP1 were deleted (Figure 1(a)). There was a good corelation between the decrease in leucine oxidation and the decrease in uptake (Figure 1(b)) when normalized to L-[U-^14^C]leucine oxidation and uptake, respectively, in the appropriate strain. These results indicate that the rate of leucine oxidation is correlated to the uptake during catabolite derepression. 

The *α*-ketoglutarate/glutamate oxido-reductive deamination reaction catalysed by glutamate dehydrogenase plays a key role in nitrogen flow and hence amino acid carbon entry into oxidative metabolism. Concomitant with the increase in leucine oxidation we also observed a significant increase in intracellular glutamate concentration in galactose grown yeast (glucose 4.5 ± 0.25 and galactose 38.2 ± 4.9, *n* = 3, *P* < 0.05).

**Figure 2:**
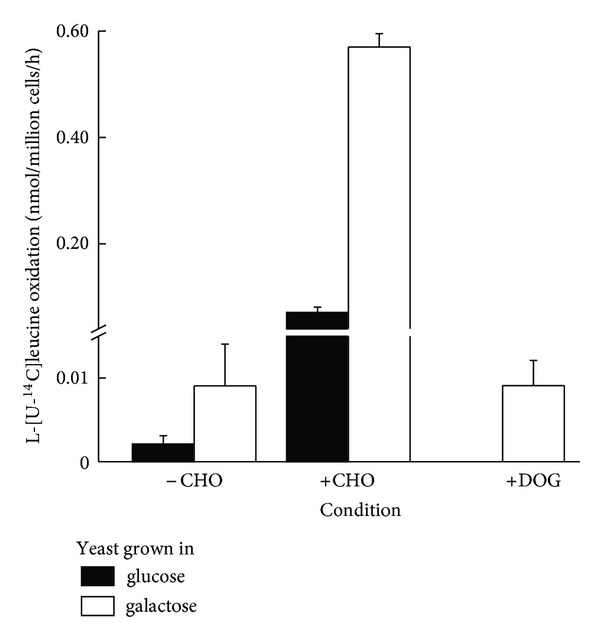
Presence of glucose or galactose in the medium is essential to measure L-[^14^C]leucine (750 *μ*M) oxidation in WT (M3750) yeast. Yeast was grown in 2% glucose (filled bars) or 2% galactose (hollow bars) and incubated in the assay medium with glucose, galactose (± carbohydrate, CHO), or the nonmetabolizable glucose analogue deoxyglucose (DOG) as described in experimental procedures. (Oxidation in yeast grown in glucose in the presence of DOG was not measured.) Results are means ± SEM for 3 different preparations measured in quadruplicate.

An interesting corollary is that in WT yeast ^14^CO_2_ production from L-[U-^14^C]leucine is dependent upon the presence of glucose or galactose (Figure 2). Yeast incubated with L-[U-^14^C]leucine but without a carbohydrate source (–CHO) released only nominal amounts of ^14^CO_2_. Addition of glucose or galactose to yeast from either ± CCR cultures (+CHO) resulted in measurable leucine oxidation (Figure 2). Deoxyglucose (+DOG) did not mimic this response indicating the requirement for a metabolizable carbohydrate source to achieve measurable leucine oxidation. 

**Figure 3:**
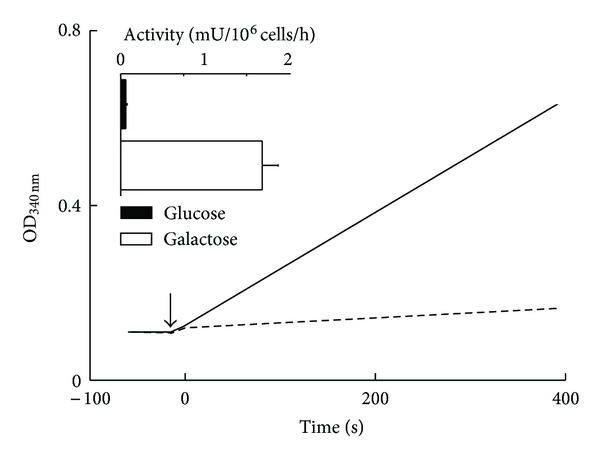
Time course of BckDH basal activity in WT yeast, grown in 2% glucose (dashed line) or 2% galactose (solid line). Representative trace of NADH formation in cell homogenates using *α*-ketoisovalerate as a BCKD substrate (added at arrow). Dashed line represents glucose and solid line represents galactose grown yeast. Inset represents mean calculated activity (*n* = 4) in mU (nmoles NADH formed/min). BckDH activity in yeast grown in glucose YNB medium (filled bars) or galactose YNB medium (hollow bars), *n* = 3.

Transamination of leucine results in the formation of the branched chain keto acid, *α*-ketoisocaproate (*α*-KIC). The key regulatory enzyme controlling entry of keto acids into oxidative metabolism is BckDH. The basal activity of BckDH was measured in whole cell extracts (after 0.05% treatment with Triton X-100 to release the enzyme from mitochondria) of WT yeast grown in 2% glucose or galactose medium; the BckDH activity determined was basal rather than total (dephosphorylated). The activity of BckDH was linear for at least 6 min (Figure 3). There was approximately a 30-fold increase in the basal BckDH activity in yeast grown in 2% galactose compared to those grown in glucose.

BckDH is a member of the 2-oxo acid dehydrogenase multienzyme complexes that contains dihydrolipoamide dehydrogenase (Lpd1). An increase in protein expression, measured by fluorescence microscopy, for Lpd1 was also observed. Analysis of images (not shown) from Lpd1-GFP strains indicates a 3.0 ± 0.5-fold increase Lpd1 expression in yeast grown in galactose compared to those grown in glucose. These results indicate that leucine catabolic pathways are upregulated during catabolite derepression. 

**Figure 4:**
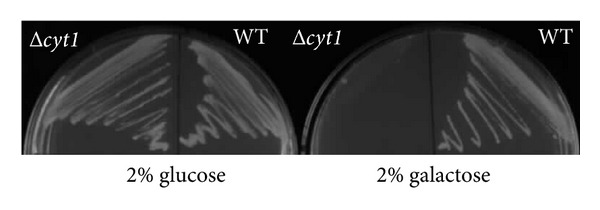
Activity of the TCA cycle is essential when WT yeast is grown with galactose as sole carbon source. Image of WT (BY4741) and Dcyt1 grown on agar plates with 2% glucose or galactose added to the YNB medium.

**Figure 5:**
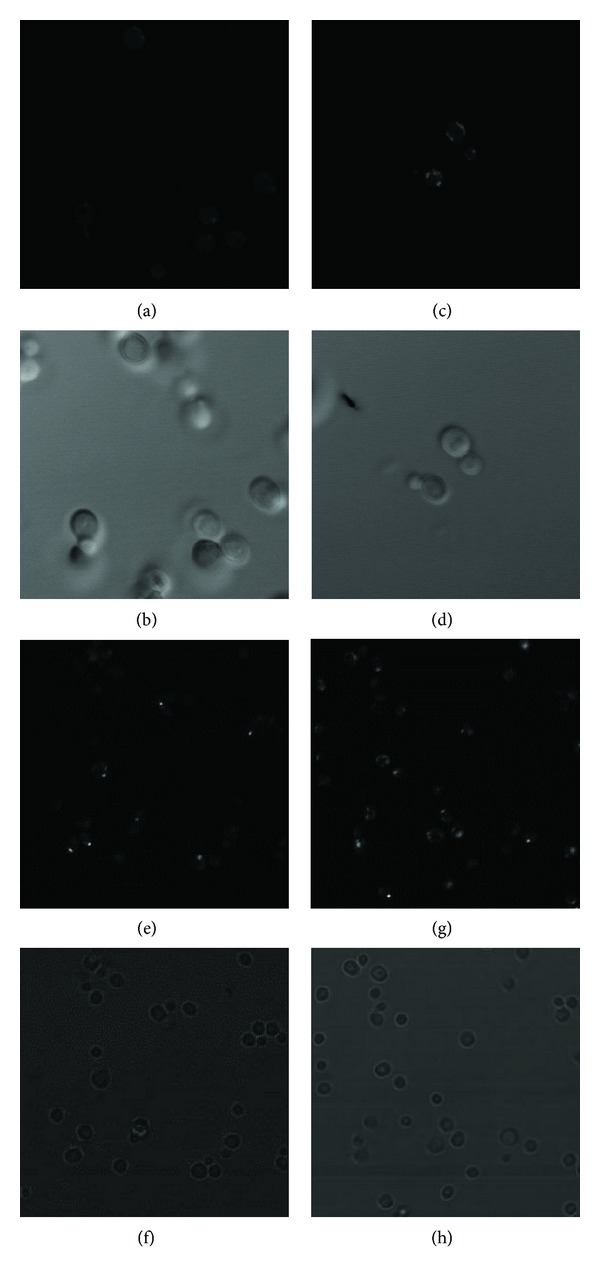
Direct fluorescence was measured in yeast strains with GFP-tagged CYT1 and CIT1 using Zeiss LSM510 and Bio-Rad Radiance 2100 confocal systems, respectively. (a) Cyt1-GFP fluorescence in yeast grown in 2% glucose YNB compared to (c) in 2% galactose YNB medium. Micrographs (b) and (d) are corresponding Nomarski differential interference contrast images of (a) and (c), respectively. (e) Cit1-GFP fluorescence of yeast grown in 2% glucose YNB compared to (g) grown in 2% galactose YNB medium. (f) and (h) are corresponding bright-field images of (e) and (g), respectively. Representative pictures of experiments in triplicate are presented.

The yeast grown with galactose as sole carbon source is in an oxidative state of metabolism with increased oxygen consumption [5]. To confirm if the yeast relied upon the activity of TCA cycle during catabolite derepression for growth and that any residual fermentative component of metabolism did not account for growth we used a strain with the cytochrome c1 gene deletion. Cyt1 is a subunit of complex III in the mitochondrial electron transport chain. There was no regression in growth of Δ*cyt1* strain compared to WT when grown in medium with 2% glucose (Figure 4). However, there was no growth visible for the Δ*cyt1* yeast when grown in galactose medium (Figure 4), even after 72 hours of incubation. Similar results were observed for growth in liquid medium (data not shown). These results indicate that reductive substrate-electron transfer is critical for growth with galactose as the sole carbon source. We further investigated the role of the TCA cycle and electron transport by examining the expression of citrate synthase and Cyt1 from the Yeast-GFP clone collection [10] in yeast grown in 2% glucose or galactose medium. The Yeast-GFP clone collection is designed to express full-length proteins, tagged at the carboxy-terminal end with GFP, from their endogenous promoters. A very faint GFP signal was observed for Cyt1 protein in yeast grown in 2% glucose (Figure 5(a)). This expression was increased by 2.0 ± 0.24-fold when WT yeast was grown in 2% galactose YNB (Figure 5(c)). Cs1 expression was visible in WT yeast grown in 2% glucose medium (Figure 5(e)); however this was significantly (*P* < 0.05, *n* = 20) increased by 30 ± 5% in yeast grown in galactose medium (Figure 5(g)). 

## 4. Discussion

### 4.1. Coordination of Nitrogen and Carbon Metabolism

Catabolite derepression, from a genetic point of view, results in transcription of numerous genes, by upstream activation sites (UAS) such as CCAAT box, R box, or CSRE element which are known to be involved in oxidative metabolic pathways [17] or PKA pathway [18, 19], and from a metabolic and functional point of view results in enhanced uptake of amino acids via TOR signal transduction [5]. Our results indicate a level of coordination between the genes activated during catabolite derepression via the UAS and via signal transduction through TOR, although the precise nature of this coordination remains an outstanding question. 

In this paper we have investigated the molecular mechanisms underlying the integration of nitrogen and carbon metabolism in *S. cerevisiae* during conditions of stress. We have demonstrated that amino acid transport and oxidation, via the TCA cycle, play a vital role in yeast survival and growth during catabolite derepression. With regards to amino acid metabolism, we propose that during catabolite derepression, the following three major steps involving genetic and metabolic reprogramming occur: (1) TOR mediated upregulation of amino acid permeases, (2) increase in the uptake of amino acids, and (3) increase in the capacity of the oxidative metabolic machinery. This model suggests that catabolite derepression is modulated via the TOR signalling pathway, which in turn increases the transcription of specific permease genes to induce permease protein expression. This allows the cell to utilise the extracellular amino acids which are fed into an already activated TCA cycle via key enzymes such as 2-oxo acid dehydrogenase multienzyme complexes and consumed for energy production.

### 4.2. Metabolic Fate of Increased Amino Acid Intake during Catabolite Derepression

Amino acids commonly provide a carbon source for entry into the TCA cycle beyond the point of 2-carbon incorporation at the citrate synthase step. Some of the amino acids whose uptake is increased during catabolite derepression by TOR mediated signalling, such as leucine, are common intermediates of major catabolic pathways such as acetyl CoA, pyruvate, or 2-oxoglutarate. Subsequent entry into intermediary metabolism, with concurrent mitochondrial electron transfer is the main route by which cellular energy is utilized from amino acids. This is achieved either by being an anaplerotic source in a complete TCA cycle, or as a direct bioenergetic substrate in a truncated cycle [20]. As a result, to achieve complete oxidation (CO_2_ release from all carbons) a source of acetyl CoA is required. In this respect, the only known pathway for the terminal oxidation of leucine is through acetoacetate to acetyl-CoA and hence subsequent oxidation in the TCA cycle. 

We have also observed, for the first time, an absolute requirement of a carbon source (e.g., glucose or galactose), *in situ*, for the increased oxidative metabolism of leucine to take place. It is particularly intriguing as the presence of carbohydrate is not necessary for the increase in L-[U-^14^C]leucine uptake observed during catabolite derepression (Figure 1). It is also not essential at the first irreversible (and rate limiting) step in leucine oxidation, that is, the activity of BckDH multienzyme complex (Figure 5), in cell extracts. BckDH oxidatively decarboxylates *α*-KIC to form isovaleryl-CoA and allows entry of the carbon skeleton of the increased cellular leucine into the TCA cycle. It is therefore likely that during catabolite derepression, an alternative nonamino acid carbon source is required for the entry of leucine into the TCA cycle downstream of BckDH. Alternatively it may be a strict requirement *in situ*; a possibility that remains to be investigated. It must be noted that under the O/N culture conditions generally employed for yeast growth in the laboratory and those used in our study, there is a significant amount of carbohydrate present in the growth medium. 

We believe the integration of amino acid transport with oxidative metabolism, demonstrated here at the molecular level, is an important advance in our understanding of intermediary metabolism, particularly the role amino acids may play in contributing to the overall balance of carbon provision for cell maintenance, growth, and proliferation during conditions of stress. Our demonstration that amino acid transport and subsequent catabolism contribute to oxidative energy supply during catabolite derepression also highlights the importance of mitochondrial redox in this control. The resultant changes to mitochondrial redox (NAD(P)^+^/NAD(P)H), evoked by both catabolism and oxidative deamination of ketoacid derived amino acid, has clear implications in other cellular processes such as modulation of histone deacetylase [4] and life span in *S. cerevisiae*.

## Supplementary Material

Supplementary Table 1: Genotype of amino acid permease deleted yeast strains.
