# Supplementary material for: Metabolic Fate of the Increased Yeast Amino Acid Uptake Subsequent to Catabolite Derepression
Source: J Amino Acids. 2013 Feb 4;2013:461901. doi: 10.1155/2013/461901 (PMC3575661; doi:10.1155/2013/461901)
Supplement: Supplementary file 1 — Supplementary Table 1: Genotype of amino acid permease deleted yeast strains. [file 461901.f1.pdf]

Table 1 Genotype of amino acid permease deleted yeast strains

M3750	*MA*Ta *ura3*
M4055	*MA*Ta *ura3 gap*1Δ Δ(*bap2-tat*1)
M4581	*MA*Ta *ura3 gap*1Δ *agp*1Δ *gnp*1Δ Δ(*bap2-tat*1)
M4582	*MA*Ta *ura3 gap*1Δ Δ(*bap2-tat*1) *bap*3Δ *tat*2Δ

All strains detailed were *MATa* and *ura3* deleted.
